# Supplementary material for: Patterns of Intron Gain and Loss in Fungi
Source: PLoS Biol. 2004 Nov 30;2(12):e422. doi: 10.1371/journal.pbio.0020422 (PMC532390; doi:10.1371/journal.pbio.0020422)
Supplement: Table S1 — Also available at http://genes.mit.edu/NielsenEtAl/. (4.3 MB ZIP). [file pbio.0020422.st001.zip › NielsenEtAl/html/716.html]

AN0472.1.NCU07076.1.MG01001.1.FG08757.1


```
 CLUSTAL W (1.82) Multiple Sequence Alignments - Introns Inserted


Sequence 1: MG01001.1	918 aa
Sequence 2: FG08757.1	850 aa
Sequence 3: AN0472.1	934 aa
Sequence 4: NCU07076.1	912 aa
Alignment Length: 1034 aa
Number Identitical Residues: 197 aa
Alignment Score (without introns) 11969


MG01001.1 	------MRFNIILTMLVGV-----ASCSSDGSENRHQHRHPHAAPAAHPAKRQVAD~SAP
NCU07076.1	--------------------------------------------------------~---
FG08757.1 	------MRSRNILPLWL-----------------------LWLTQGQVSALPQVDQ~DSS
AN0472.1  	MEAKCKTRLANVLMLLLAARTVCSAPTPSQSSGVSESTQYPPSTRGARLLPPETQG0FYP
          	 .:...           .  : .::. .:..:    .      : .      .      .

MG01001.1 	GARSTTPPQSVVASPTGSSPVTRPANEK-PKATSVGVLITELEG-GVAQIITARPGPPPD
NCU07076.1	------------------------------------------MAVTTPTVAPSPSYSCPS
FG08757.1 	NSQNDEVIGDIQTSAFEYPTTYAKEDII-PVTETVKVSTSKVGTTATAQVLPSLSDVEPH
AN0472.1  	TKLEREGHELEQPTPTPDESFVGLNDLLDTLGQPESLLNWLLPNPDEPTDVPSQPPAAPT
          	   .        .:.    .     .  ..   .             .   .: .   * 

MG01001.1 	TKGTVITKLNTTGHATIAPLLTRPIPPVKERFEVDLQARSAAAIATRNPSPSRPAVARMA
NCU07076.1	CKGTGS---------KPSPVHSSSSSPFPS-------KGYSQQSPPKNKRNNNNSTSKRS
FG08757.1 	TEGFIVTDLYNP-FFTKPAVLSTSIPSPTS-------LLKPVKTTTKNAEPSG-LSKMAA
AN0472.1  	SEASSTPLVAATPVPTTLPATHNIIEQPTTVSSVPSSFEATTVTTSSSDENSPVVSTFTT
          	 :.   .    .   .  .             .   .   .   .. .   .       :

MG01001.1 	APNI~FAEPISTN-PPHAKFLRRDDHPVGRSSI~TSL-----APYPTNKFFSNMYLGGMR
NCU07076.1	DGDL~FSTPISPELSIPKSIPSQPNHPAPRKGI~KQSQEGGGTAIPTNKFHANFFLGSQS
FG08757.1 	AADI~FASPIDTG-APLSMFARKDTHPVSKDAV~KQN-----SPIGTNKFYSNFYLEDQN
AN0472.1  	HIQD1VFVPVGTG-PIPAAITSRNDHPVRKNGV0NSS-----NPIETNKFHSALFLGTQT
          	  :  .  *:..  .    :  :  **. :..: ..       .  ****.: ::*    

MG01001.1 	SPAFLHPYSIQWAKGEGVTGSWGFAISHTEPNQRVFGPPDP--KTKASKYFINPIGIHSI
NCU07076.1	STVWTHPYSLQWAKGQGSSGAWGLAVSHADASQRVFGEQDPN-GSGAARWFGSPVGRADV
FG08757.1 	TPVYTYPYSLTWAKGTGPSASWGMSISHIDADKRTYGPVG---ANGAVEFYTNPVSIQPM
AN0472.1  	NATFTHPYTLAWAKGSGNLSSFGMAVSHTELNLVASGPTNSQLPGNPISYYINPIGIQSI
          	...: :**:: **** *  .::*:::** : .  . *  ...    .  :: .*:.   :

MG01001.1 	VLSAQELGPQTQLAMTELCAFSAMAE---LRATPGSQPTLKMPFVQGSGFITGIYNGASP
NCU07076.1	VLSEQGLEKGNATGGVELMTQDVKEQSIRVQLVTGGDVKLEAPVVQGMGMVTALYKQGTP
FG08757.1 	VISAKELGKNTVLTMDSISPHFAKAY---LRKDSKSAATITFPLYQGSVFTTAYFSGGTP
AN0472.1  	ILSASEFGPSTVLTAENPKSFSADAV---LQPQAGSARRLTLPVLQGMAFVTGIYTQLQP
          	::* . :   .     .  .  .      ::  . .   :  *. **  : *. :.   *

MG01001.1 	IVSSGVYFRQVLRANQNPQPGVVKYKMFLEDGKSWLLYARH------TSGNP--LILDVV
NCU07076.1	VIKSGVGWKMVTQAMGVVKTGVVKYRLVMEDGATWLLYATGGDVGAIKRGEPGGLELEVQ
FG08757.1 	WIQSGVYFRTMTQVTKDPKTNVRKYNFVLEDGTTWRLYAYK------TKGDP--LTLQVT
AN0472.1  	VVQSAVHFRQVVTAG-SPRAGIFKYRATLEDNSIWLIYAIP------DNGQD--PNFQLV
          	 :.*.* :: :  .    :..: **.  :**.  * :**          *:     ::: 

MG01001.1 	DSKNFKAREPFWGTIQIAKEP--GNGEA-------IYDQACGAYATSMELSGTTSGTSGS
NCU07076.1	GNNVAVAKGSFTGMVQVAKLPGGANGTEGVADAEKLYDAACGTYATGVELSGSVDGQAGR
FG08757.1 	NNGLAVSQKPFYGHIQIAKDPKVGQSEQ-------VLDNGCGIYATGMTISGNNVGTQGT
AN0472.1  	STTDLRGPSGWSGTVQVAKNPAGSSGES-------LFDNSAGVFAVEATVSGSVDGHTGT
          	..    .   : * :*:** *  ...         : * ..* :*.   :**.  *  * 

MG01001.1 	YTFTFKRAGLASS--KLLMFALPHHADGFSEASRQAVTG-LQLQTTTKGLASGILADSWT
NCU07076.1	YTFNFQKEGLSTN-SSLVMFALPHHQASFSADTASKVVSSVKLDTTTKGVAVAVVGDQWT
FG08757.1 	YKFTWTKAGHATG--KLWIFALPHHISSFDAATTKEMRT-YSMQTLTKGVAKAVGGNSWT
AN0472.1  	YRLAWAKAGKETQSTPLMMFALPHHVASFDSQTSARAVN-ITLRTTTKGMARAVIGEYWT
          	* : : : *  : :: * :******  .*.  :         : * ***:* .: .: **

MG01001.1 	IRETL-TPG-VGFLPWDRRRGEISKLGNSARIAVLRIAQQELA-----QNMIDQANLDSM
NCU07076.1	MEEDVSKPMSMGFVPWTPETGSVKTISDSAKEYIKTVAQKELLGTSQGQDILNQTDQPSM
FG08757.1 	MIEPS-LPVNMGFAPWDPVKG-SRGLSANAKKIIAPIAKSEVS-----QDMDAQSNTDSM
AN0472.1  	MTEPD-LPTNMGFAPWVLATDSSPQLSAAAQAVIRDVAITELQ-----QDIDGQTNLNSM
          	: *    * .:** **    ..   :.  *:  :  :*  *:      *::  *::  **

MG01001.1 	YFSGK0ALAKFASIIIVANDLLKNPEVAQTGLNQLKRAFAIFAENRQKFPLVYDT~RWGG
NCU07076.1	YFGGK~ALAKFAAILVAVNDVLGETDMAKKGLEQLKVAFARYSENQQQYPLVYDQ1SWGG
FG08757.1 	YFSGK0ALAKFGSVVYVINNMLGDTALAQAGLEKLKTAFARFGSNKQKFPLVYES1VWGG
AN0472.1  	YFSGK~ALSKFATIIWTVDKLLNDHATAAPALERLKQAFARFAQNKQQFPLVYDT~VWKG
          	**.** **:**.::: . :.:* :   *  .*::** *** :..*:*::****:   * *

MG01001.1 	IVSSAS2SSS-LTITHPGNTYYNDHHFHYGYFVYTAAVIAHIDKAWG--NANKEFVNTLV
NCU07076.1	IVSSAS~YTTGDAGVDFGNTYYNDHHFHYGYFIYTGAVLAHLDPSWASTASNLAYVNSLV
FG08757.1 	IVSTAS~YVSGSAGSDFGNTYYNDHHFHFGYHVLTAAYIGSMDSKWL--AANKAYVNTLV
AN0472.1  	VVSSAS~YGG-DVGADFGNTLYNDHHFHYGYFIHAAAVIGALDPSWI--AANRDWVNTLV
          	:**:**      .  . *** *******:**.: :.* :. :*  *    :*  :**:**

MG01001.1 	RDFANPSA-KDGFFTMYRNFDWYHGHSWAHGLYDTLDGN0DQESSSEDAMSAYAVKMWGR
NCU07076.1	RDVANPSSSLDPHFPSFRTFDWYHGHSWAHGLFESSDGK~DQESSSEDSMHVYALLMWAQ
FG08757.1 	RDYANPSS-SDKYFPQWRSFDWYHGHSWAHGLTPMWDGK~DQESSSEDMMSVYAMKMWGT
AN0472.1  	RDSGNPAY-NDPLFPFSRAFDWYHGHSWAKGLFESFDGK~DQESSSEDSMYAYALKMWGK
          	** .**:   *  *.  * **********:**    **: ******** * .**: **. 

MG01001.1 	AIGDANMEAR~GELQLAAQARAFKYYYLYTSDN---KVQPAEFVGNKVAGI~LFENKIDH
NCU07076.1	ATNNQALYQR~SALQLSLLSRSLNSYYLYSSSNPSSQIQPKEFVGNKVAGI~LFENKIDH
FG08757.1 	VIKDTNMVAR2ANLQLAVMSRAMQAYYYYTTTN---TVQPKNFIGNKVAGI~LFENKVHH
AN0472.1  	TIGDASMEAR~GNLMLGIMRRSFHDYFLMESDN---ANHPANFIGNKVTGI0LFENKVDH
          	.  :  :  * . * *.   *::: *:   : *     :* :*:****:** *****:.*

MG01001.1 	TTFFGANID2----~---------------DQFVREEWATYFDN---GRINEVQGGWRGI
NCU07076.1	VTFFGNKQE~YIQG~IHMLPLMPHTLFVRNREFVKEEWEAFFAG---GAAEKAEGGWKGV
FG08757.1 	TTWFSADIE~AVQG~IHMIPILPFSNLARTGTFVQQEWETYFNK---GRVDKFNNLWKGI
AN0472.1  	TTYFGSNLE~YIQG2IHMLPLLPMSPYIRSQRFVREEWNAIFASNAAAPADGVTGGWKGI
          	.*:*. . :   ..     .  . :    .  **::** : *  .::.  :   . *:*:

MG01001.1 	LMGNYAQINPAMAYSFFG------------SSKFDPGFLDG-------GASRTWYMAYSA
NCU07076.1	LYGNYATIDPRGAWEVFTGGSQLVGSPGSGNGNGNGTTVTGTGTGTGTGMVESMTSTAST
FG08757.1 	IYGNYATIQPNAAWSFFT------------QSKFDPIWIDG-------GASRTWYMAYAA
AN0472.1  	LYANLALIDPAASWRFFS------------QSNFDYSWIDG-------GASRTWYLAFAA
          	: .* * *:*  :: .*             ..: :   : *       *  .:   : ::

MG01001.1 	A1LHPEYPRSPLVYHEWRLSFSALLLGRPGEAGFR0TDHEVGGGAGAEQLMGWLVAAGP-
NCU07076.1	A~STTTTPGSTTPTSTSVPTTPSAMSSKMVRRTIR~RALEKGKGKMNMPLARAALPKAKK
FG08757.1 	A1LGSI-----------------------------~------------------------
AN0472.1  	G1RITDIAGGAKLTSWNAVACASALLRDFITYTSR~------------RLLCRVVKV---
          	.   .  . ..        : .:                                     

MG01001.1 	---GVTYVTTGEAP---------~----
NCU07076.1	DGFGPENIDGGASLTWYLTWCAT1LGGL
FG08757.1 	-----------------------~----
AN0472.1  	-----------------------~----
          	
```
